# Doxorubicin-Loaded Metal-Organic Frameworks Nanoparticles with Engineered Cyclodextrin Coatings: Insights on Drug Location by Solid State NMR Spectroscopy

**DOI:** 10.3390/nano11040945

**Published:** 2021-04-08

**Authors:** Xue Li, Marianna Porcino, Jingwen Qiu, Doru Constantin, Charlotte Martineau-Corcos, Ruxandra Gref

**Affiliations:** 1 Institut des Sciences Moléculaires d’Orsay, UMR CNRS 8214, Université Paris-Sud, Université Paris Saclay, 91400 Orsay, France; xue.li@universite-paris-saclay.fr (X.L.); jingwen.qiu@universite-paris-saclay.fr (J.Q.); 2 CEMHTI UPR CNRS 3079, Université d’Orléans, 45071 Orléans, France; marianna.porcino@cnrs-orleans.fr; 3 Laboratoire de Physique des Solides, UMR 8502, Université Paris-Sud, 91405 Orsay, France; doru.constantin@universite-paris-saclay.fr; 4 ILV UMR CNRS 8180, Université de Versailles St-Quentin en Yvelines, Université Paris Saclay, 78035 Versailles, France

**Keywords:** metal-organic frameworks nanoparticles, doxorubicin, cyclodextrin, solid state NMR spectroscopy

## Abstract

Recently developed, nanoscale metal-organic frameworks (nanoMOFs) functionalized with versatile coatings are drawing special attention in the nanomedicine field. Here we show the preparation of core–shell MIL-100(Al) nanoMOFs for the delivery of the anticancer drug doxorubicin (DOX). DOX was efficiently incorporated in the MOFs and was released in a progressive manner, depending on the initial loading. Besides, the coatings were made of biodegradable γ-cyclodextrin-citrate oligomers (CD-CO) with affinity for both DOX and the MOF cores. DOX was incorporated and released faster due to its affinity for the coating material. A set of complementary solid state nuclear magnetic resonance (ssNMR) experiments including ^1^H-^1^H and ^13^C-^27^Al two-dimensional NMR, was used to gain a deep understanding on the multiple interactions involved in the MIL-100(Al) core–shell system. To do so, ^13^C-labelled shells were synthesized. This study paves the way towards a methodology to assess the nanoMOF component localization at a molecular scale and to investigate the nanoMOF physicochemical properties, which play a main role on their biological applications.

## 1. Introduction

Metal-organic frameworks (MOFs) are a class of ordered coordination network built from metal clusters as inorganic parts and organic ligands as linkers, containing potential voids [1]. Aluminum-based MOFs were among the first synthesized and studied because they are stable and highly porous. They attract a growing interest due to their “facile” hydrothermal synthesis [2]. In addition, aluminum is relatively cheap and abundant, making it an attractive metal source for mass production of MOFs. The two most reported aluminum-based MOFs are MIL (stands for materials of Institute Lavoisier) and CAU (stands for Christian–Albrechts University). Aluminum-based MOFs found useful applications in gas separation and storage, organic pollutants removal, pervaporation, and more recently, in biomedicine. Interestingly, MIL-100(Al) based metal organic gels [3] have been used as drug carriers to deliver doxorubicin (DOX). Solid-state NMR (ssNMR) spectroscopy has emerged as an essential analytical technique to achieve detailed atomic-scale characterization of complex porous systems [4], including MOFs [5,6,7,8] The technique is non-destructive, allows probing a local scale highly complementary to diffraction and is sensitive to millisecond timescale, hence can be used to monitor dynamic processes. For instance, the activation process of MIL-100(Al) could be followed by ssNMR [9]. Although more challenging, paramagnetic MOFs can also be characterized by ssNMR spectroscopy [10].

In the field of biomedicine, ssNMR is also of particular interest [5,11,12,13,14,15]. Notably, ssNMR brought valuable insights on the supramolecular structure of highly porous core–shell drug nanocarriers, namely MIL-100 nanoMOFs coated with cyclodextrin-phosphate (CD-P) molecules. The first investigation [16] was performed on paramagnetic MIL-100(Fe) nanoparticle using ^1^H solid-state magic-angle spinning (MAS) NMR analysis. The obtained ^1^H NMR spectra revealed the interaction between the nanoMOFs and the CD-P materials by showing the close proximity between the CD-P and the paramagnetic centers on the nanoMOF external surfaces. It was thus demonstrated that cooperative interactions occurred between the coating and the core, based on phosphate-iron coordination, ensuring good shell stability in biological media. However, a large number of spinning side bands were observed due to the presence of the Fe paramagnetic center, limiting the amount of information that could be extracted from the NMR spectra. More recently, studies were carried out using the diamagnetic MIL-100 (Al) coated and loaded with phosphorus-containing species [17]. High-resolution ^31^P-^27^Al two-dimensional (2D) NMR experiments clearly evidenced the interactions between the nanoMOF external surfaces and the phosphorus-containing coating molecules at the molecular level. Moreover, useful information has been obtained on the interactions between the nanoMOFs and the drugs located inside their micropores [17].

In the present study, we intended to go steps beyond and take advantage of the possibilities offered by ssNMR to investigate the interactions in more complex core–shell nanoMOF systems. Recently, versatile polymeric CD-based coatings were engineered to ensure a convenient one-step surface modification of MIL-100 nanoMOFs [18]. The coatings were made of biodegradable γ-CD-citrate oligomers (CD-CO), which could be functionalized by click chemistry with fluorescent dyes [19]. Here, we took advantage of the known affinity between DOX and both the biodegradable γ-CD-citrate oligomers (CD-CO) [20] and the nanoMOFs [13] to prepare dual (core–shell) DOX-loaded nanoMOFs. Indeed, DOX was expected to be loaded both in the pores of the nanoMOFs and the γ-CD cavities located in the coating layers. The interaction of the DOX molecules with both the CD-CO shell and the MIL-100 (Al) nanoMOFs core was confirmed by a set of complementary ssNMR experiments, including 2D ^1^H-^1^H and ^13^C-^27^Al NMR. To enable this study, ^13^C-labelled CD-CO oligomers were synthesized for the first time. DOX release in phosphate buffer saline (PBS) was investigated as a function of the drug payload, considering the possibility of DOX self-association inside the cages. This study focuses on a comprehensive understanding at a molecular scale of the drug localization and the physicochemical properties, which play an important role in the biological applications.

## 2. Materials and Methods

### 2.1. Materials and Reagents

Doxorubicin (DOX, 98%), citric acid (≥99.5%), sodium phosphate monobasic dihydrate (≥99%, NaH_2_PO_4_·2H_2_O), hydrochloric acid (HCl, 34–37%), sodium hydroxide (NaOH, ≥97%) potassium chloride (powder, ≥99%) and deferoxamine mesylate salt (≥92.5%) were purchased from Sigma-Aldrich (Saint-Quentin-Fallavier, France). γ-CD was purchased from Cyclolab (Budapest, Hungary). Dimethylsulfoxide (DMSO, ACS) was from Panreac Applichem (Barcelona, Spain). Deuterated DMSO (DMSO-*d*_6_, 99.8% D) and water (D_2_O, 99.9% D) were obtained from Eurisotop (Gif sur Yvette, France). 1,5-^13^C_2_ citric acid was obtained from CortecNet (Les Ulis, France). 1,3,5-benzenetricarboxylic acid (BTC, 95%, Sigma-Aldrich, Saint-Quentin-Fallavier, France), aluminum nitrate nonahydrate (98%, Sigma-Aldrich, Saint-Quentin-Fallavier, France) and trimethyl trimesate (98%, Sigma-Aldrich, Saint-Quentin-Fallavier, France) and absolute ethanol (99%, Carlo Erba, Val-de-Reuil, France) were used for the synthesis of nanoMOFs. Water was purified by a Millipore MilliQ system.

Phosphate buffer saline (PBS, pH 7.4, containing 9.5 mM phosphates) was purchased from Life Technologies (Saint-Aubin, France) and was used for DOX release study.

### 2.2. Synthesis and Characterization of γ-CD-Citrate Oligomers

The γ-CD-citrate oligomers (CD-CO) were synthesized by adapting a previously reported method [21]. Briefly, the reaction mixture was prepared by solubilizing 0.09 mmol γ-CD, 0.45 mmol citric acid, and 0.2 mmol NaH_2_PO_4_.2H_2_O in 2 mL water. This solution was concentrated by evaporation at 140 °C for 10 min, followed by further heating at 140 °C for 15 min under reduced pressure (10–15 mmHg). Then 10 mL distilled water was added and the crude was sonicated for 5 min, followed by filtration to remove the insoluble fraction. The soluble fraction was dialyzed for 48 h using a cellulosic membrane (cut-off 20 kDa, Spectrum Laboratories, Piscataway, NJ, USA). Finally, the CD-CO and CD-^13^CO were obtained as a solid white powder after freeze drying. To obtain CD-^13^CO, a similar method was employed except that 1,5-^13^C_2_ citric acid was employed.

The composition of the synthesized CD-CO was determined by proton nuclear magnetic resonance (NMR) spectroscopy. Fourier-transform infrared (FTIR) spectra were recorded with a Bruker apparatus (VERTEX 70). The average molecular weight of the CD-CO and CD-^13^CO was determined by size-exclusion chromatography (SEC), coupled on-line with multiangle light scattering (MALS) and refractive index (RI) detectors (SEC/MALS/RI, Wyatt Technology, Santa Barbara, CA, USA) [19].

### 2.3. Synthesis and Characterization of MIL-100 (Al) nanoMOFs

Aluminum trimesate MIL-100 (Al) nanoMOFs were synthesized as previously described [17,22]. Briefly, 20 mL of an aqueous mixture containing aluminum nitrate nonahydrate (1.43 g) and trimethyl trimesate (1.21 g) was mixed with nitric acid (4 mL, 4 m) at room temperature and then heated at 210 °C for 30 min under stirring by microwave. The synthesized MIL-100 (Al) nanoMOFs were obtained by centrifugation at 10,000× *g* for 15 min and activated by dispersing them in methanol overnight (50 mL) with vigorous stirring. The as-synthesized -MIL 100(Al) nanoMOFs were recovered by centrifugation (10,000× *g*, 15 min).

The average hydrodynamic diameters and size distributions of the nanoMOFs were determined by dynamic light scattering (DLS, Malvern Nano-ZS, Zetasizer Nano series, Orsay, France). The morphology of the nanoMOFs was investigated by transmission electron microscopy (TEM, JEOL 1400 (120 kV), Jeol Ltd., Tokyo, Japan). Powder X-ray diffraction patterns (PXRD) were recorded to characterize the crystallinity. The nanoMOFs BET (Brunauer–Emmett–Teller) surface area was measured by nitrogen sorption experiments at −196 °C using an ASAP 2020 (Micromeritics, Norcross, GA, USA) after degassing at 100 °C for 15 h under secondary vacuum. FTIR spectrum was recorded. NanoMOFs were stored in ethanol and redispersed in aqueous media before usage.

### 2.4. DOX Encapsulation in nanoMOFs Coated or Not with CD-CO

MIL-100(Al) nanoMOFs ethanolic suspensions were first centrifuged at 10,000× *g* for 10 min to recover the nanoMOF pellets, which were further redispersed in water. For DOX encapsulation, 1 mL of nanoMOFs aqueous suspension (2 mg/mL) was mixed with 1 mL of DOX solution (0–2 mg/mL), followed by gently stirring for 1–6 days at room temperature. Theoretical drug loading (TDL), calculated as the weight ratio (%) between DOX and nanoMOFs, was in the range of 10–100%.

The same procedure was used to load DOX in MIL-100(Al) nanoMOFs coated with CD-CO (CD-CO@ MIL-100 (Al)), where the weight ratio between CD-CO and nanoMOFs was kept as 1:2 and CD-CO was added at the same time as DOX.

The DOX loaded nanoMOFs were recovered by centrifugation (10,000× *g*, 10 min) and the supernatant was used to quantify the drug payload, as according to Equation (1):

(1) $$Payload\;\left(\%\right)=\frac{Encapsulated\;drug\;\left(\mathrm{mg}\right)}{Empty\;nanoMOFs\;\left(\mathrm{mg}\right)}$$

taking into account the amount of nanoMOFs (mg) used for encapsulation and the amount of DOX incorporated at the end of the incubation.

Encapsulation efficiency (*EE*) was calculated, as according to Equation (2):

(2) $$EE\;\left(\%\right)=\frac{\mathrm{Encapsulated}\;\mathrm{drug}\;\left(\mathrm{mg}\right)}{\mathrm{Initial}\;\mathrm{Drug}\;\left(\mathrm{mg}\right)}\times 100$$

DOX was quantified by fluorescence spectroscopy with excitation (λ_ex_) and emission (λ_em_) at 480 and 590 nm, respectively. To do so, DOX in the nanoMOF supernatants was diluted with a mixture of water and DMSO (1:1 *v*:*v*), to dissociate the complexes DOX:CD before the quantification. The same loading and quantification methods were used for MIL-100 (Al) nanoMOFs, coated or not with CD-CO.

### 2.5. Characterization of CD-CO Coated nanoMOFs

The morphologies of nanoMOFs, loaded or not with DOX, coated or not with CD-CO, were observed using TEM. Their crystallinity was characterized by PXRD using a homemade setup based on a copper rotating anode generator (RU-200BEH, Rigaku Ltd., Tokyo, Japan), as previously described [18,23,24,25]. The accessible scattering vector range was 0.035–0.5 Å^−1^.

Colloidal stabilities of DOX loaded nanoMOFs, coated or not with CD-CO were estimated by DLS after incubation in MilliQ water. Zeta potential (ZP) of the same nanoMOF series were measured at 25 °C using a Zetasizer instrument (Malvern Nano-ZS, Zetasizer Nano series, France) in a pH range of 1–10, adjusted using HCl or NaOH. For the measurements, nanoMOFs were diluted using a KCl solution (1 mM) to a final concentration of 100 µg/mL.

### 2.6. DOX Release Study

The DOX release experiments were carried out by dispersing DOX loaded nanoMOFs in PBS to reach a final nanoMOF concentration of 100 µg/mL, followed by incubation at 37 °C for 30 days under gentle shaking. The release profile was obtained by measuring both the DOX concentration in the supernatant and in the pellet at periodic time intervals. Supernatants were obtained after centrifugation at 17,000× *g* for 20 min, followed by dilution with a mixture of water and DMSO (1:1 *v*:*v*) and analyzed by fluorescence spectroscopy to determine the DOX concentrations. DOX in the pellet was extracted by degrading the DOX-loaded nanoMOFs (100 µg/mL) in 10 mg/mL of deferoxamine mesylate salt after 24 h incubation at 37 °C. The extracted DOX was further diluted with a mixture of water and DMSO (1:1 *v*:*v*) and quantified by fluorescence spectroscopy.

### 2.7. Solid-State NMR Spectroscopy

The ^1^H, ^13^C, and ^27^Al MAS NMR spectra were recorded at a magnetic field of 9.4 T, using a Bruker 400 MHz WB NMR spectrometer and a HX 4 mm probe. The ^1^H spectra were acquired using a Hahn echo pulse sequence, with a 90° pulse duration of 3 µs, an interpulse delay synchronized with one rotor period and a spinning rate of 10 kHz. The recycle delay was set to 3 s and 32 transients were recorded for each sample. The ^13^C cross-polarization under MAS (CP-MAS) spectra were recorded with a contact time of 3.5 ms, a recycle delay of 3 s, and the initial 90 pulse on ^1^H to 3 µs with a radio frequency (RF) field of 80 kHz. ^1^H SPINAL-64 decoupling was applied during the ^13^C acquisition. The ^1^H and ^13^C chemical shifts were referenced to Adamantane. For the ^27^Al MAS NMR, the recycle delay was set to 0.3 s. The ^27^Al chemical shifts are referenced to Al(NO_3_)_3_ solution at 0 ppm.

The ^13^C {^27^Al} symmetry-based resonance-echo saturation-pulse double-resonance (S-RESPDOR) [22,26,27,28,29] experiments and ^27^Al{^13^C} dipolar based heteronuclear multiple quantum correlation (*D*-HMQC) 2D experiments were performed under a MAS frequency of 12.5 kHz in a 4 mm probe using the same spectrometer mentioned before and a REDOR box [30]. SR4^2^_1_ [31,32] was used as the recoupling sequence in order to reintroduce ^13^C-^27^Al heteronuclear dipolar interactions, with different recoupling time. Recycling delay of 2.5 s was used and 8192 transients were recorded for each CP CP-RESPDOR experiment. The RF field of ^27^Al saturation was around 85 kHz. For the HMQC, 40 t_1_ slices with 6528 transients were co-added, leading to a total of experiment time of around 21 h. The states procedure provides a phase sensitive 2D NMR spectrum. All spectra were treated with 100 Hz exponential apodization in both dimensions.

The ^1^H MAS and ^1^H-^1^H 2D MAS exchange spectroscopy (EXSY) NMR experiments were recorded on the spectrometer mentioned before and using HXY 1.3 mm probe, with a recycle delay of 3 s and a ^1^H 90 pulse of 1.1 µs. Two different mixing times were used for the exchange experiments (5 ms and 15 ms) and 250 t_1_ slices with 128 transients were coadded. The states procedure provided a phase sensitive 2D NMR spectrum. The samples were dried under vacuum at 40 °C before the experiments.

The ^1^H{^13^C} *D*-HMQC NMR spectra were recorded at 17.6 T using a 1.3 HXY probe in a double mode with a spinning rate of 50 kHz. The symmetry-based SR4^2^_1_ scheme was used to recouple the ^1^H-^13^C dipolar interaction at different recoupling times and a RF field of 100 kHz. Recycling delay was set at 3 s, with 320 transients, leading to a total experimental time of 15 min each.

All the samples were finely ground into powders and packed into a zirconia rotor of the appropriate size. The NMR spectra were acquired using TopSpin 3.5 Bruker Software (Bruker BioSpin GmbH, Karlsruhe, Germany) and processed with DmFit software [33].

## 3. Results

### 3.1. DOX Encapsulation in nanoMIL-100(Al)

**Figure 1:**
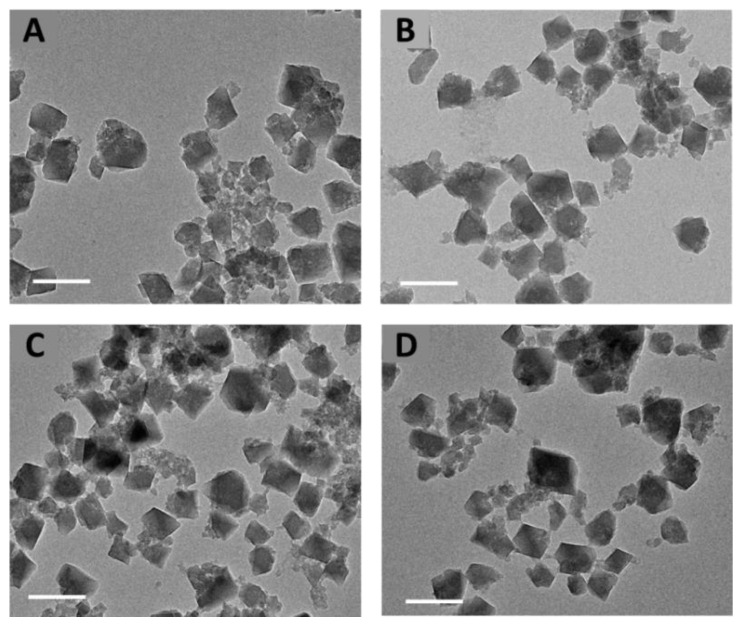
TEM images of MIL-100 (Al) nanoMOFs (**A**), CD-CO@ MIL-100 (Al) (**B**), DOX loaded MIL-100 (Al) (**C**) and DOX loaded CD-CO@MIL-100 (Al) (**D**) (scale bar: 200 nm).

MIL-100(Al) nanoMOFs with mean hydrodynamic diameters of 260 ± 8 nm were successfully synthesized. They exhibited facetted morphology (Figure 1A) and a crystalline structure according to TEM and PXRD investigations, respectively. The Brunauer–Emmett–Teller (BET) surface area reached 1720 ± 65 m^2^/g. The nanoMOFs displayed a positive surface charge characterized by a Zeta potential of 10 ± 4 mV, and composition in agreement with previously reported data [22].

**Figure 2:**
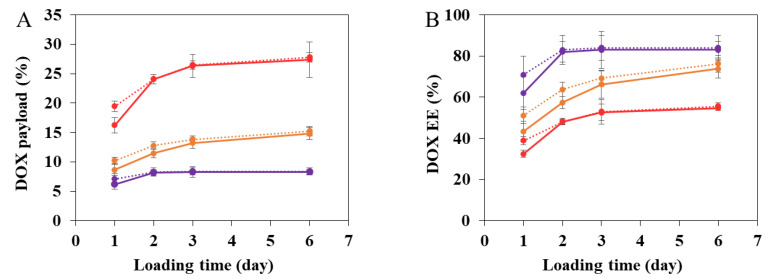
DOX payload (**A**) and encapsulation efficiency (EE, (**B**)) in MIL-100 (Al) nanoMOFs (continuous line) and nanoMOFs coated with CD-CO (CD-CO@nanoMOFs, dotted line) at different theoretical drug loading (TDL) (purple: 10%; orange: 20%; red: 50%).

Firstly, DOX incorporation was carried out by simply incubating the nanoMOFs in aqueous DOX solutions under rotative agitation, at room temperature for 1–6 days. Figure 2 reports the kinetics of DOX encapsulation obtained by fluorescence spectroscopy. DOX incorporation kinetics were investigated at different DOX initial amounts calculated as the TDL, which is the weight ratio between DOX and nanoMOFs used in the loading process. When TDL was 10%, the DOX encapsulation efficiency (EE) reached 62.5 ± 17.5% within 1 day and plateaued in 2 days at 83.6% ± 7.4% (corresponding to a DOX payload of 8.3 ± 0.7 wt.%). At a TDL of 20%, the EE plateaued at 73.9% ± 4.2% (corresponding to a DOX payload of 14.8 ± 0.8 wt.%). At TDL of 50%, DOX payload increased to 27.4 ± 2.0 wt.% after 6 days’ impregnation. This clearly shows that DOX efficiently interacted with MIL-100 nanoMOFs. In contrast, DOX payload in other type of nanoparticles (NPs) such as polymeric NPs is not as high. For example, DOX payload in poly lactic-co-glycolic acid (PLGA) NPs was less than 15 wt.%, whatever the preparation method [34]. Moreover, DOX payload in the commercial Doxil^®^ (doxorubicin loaded liposome) was around 12 wt.% [35].

DOX loading in MIL-100 nanoMOFs (Fe) was also investigated [13] showing DOX and nanoMOFs association constant Ka (1:1) of (1.1 ± 0.1) × 10^4^ M^−1^. The maximal DOX payload in MIL-100 (Fe) nanoMOFs was reported as 32.5 wt.% [36] at TDL of 150% after 24 h incubation, which is similar to what is observed in this study. DOX loading in MIL-100 (Al) metal organic gels (MOGs) [3] was reported to be 620 mg/g, however, no studies were carried out to investigate whether DOX was well entrapped inside the MIL-100 (Al) porosity, or if it was located on the nanoMOF surface or inside the gel network.

**Figure 3:**
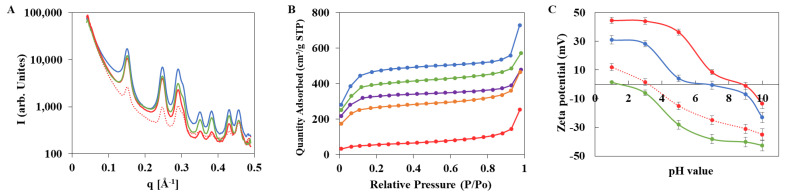
Characterization by PXRD (**A**), porosimetry (**B**), and Zeta potential (**C**) of the particles before and after drug loading and/or surface functionalization (black: simulated PXRD patterns for MIL-100 (Al); blue: empty nanoMOFs; green: nanoMOFs coated with CD-CO; purple: nanoMOFs loaded with DOX at TDL of 10%; orange: DOX at TDL of 20%; red: DOX at TDL of 50%; red dashed line: nanoMOFs loaded with DOX at TDL of 50% and coated with CD-CO).

Noteworthy, the nanoMOFs maintained the same morphology with faceted-type structures before and after DOX encapsulation, whatever the payloads, as shown by TEM images (Figure 1C). The colloidal stability of nanoMOFs before and after drug loading was investigated. After DOX loading in nanoMOFs by impregnation, no aggregation was observed, whatever the TDL, 20% or 50%. PXRD studies showed that the crystalline structure of the MIL-100 (Al) nanoMOFs was not affected upon DOX loading (Figure 3A). However, at the highest DOX payloads, the BET surface area was dramatically reduced (1720 ± 65 m^2^/g, 1245 ± 80 m^2^/g, 1010 ± 65 m^2^/g, and 200 ± 35 m^2^/g, for nanoMOFs loaded with 0%, 8.3 ± 0.7 wt.%, 14.8 ± 0.8 wt.%, and 27.4 ± 2.0 wt.% DOX, respectively (Figure 3B), suggesting that DOX localized inside the pores of the nanoMOFs. The presence of DOX is also confirmed by FTIR, showing the DOX peaks between 3000 and 3700 cm^−1^ on the DOX loaded particles (Figure S1).

To gain insights on the effect of DOX loading and CD-CO coating on the surface properties of nanoMOFs, Zeta potential (ZP) was measured. In agreement with previous studies, empty and uncoated nanoMOFs exhibited positive ZP (4.2 ± 2.3 mV) at pH 5, shifting to negative values (−0.5 ± 2.0 mV) at pH 7. In contrast, at maximal DOX loading (27.4 ± 2.0 wt.%), the ZP of nanoMOFs reached increased ZP values (+4.2 ± 2.3 mV and +36.4 ± 2.1 mV for empty nanoMOFs and DOX loaded nanoMOFs at pH 5, respectively) possibly due to the cationic character of DOX [37]. This tendency of positive shift decreased in basic conditions.

**Figure 4:**
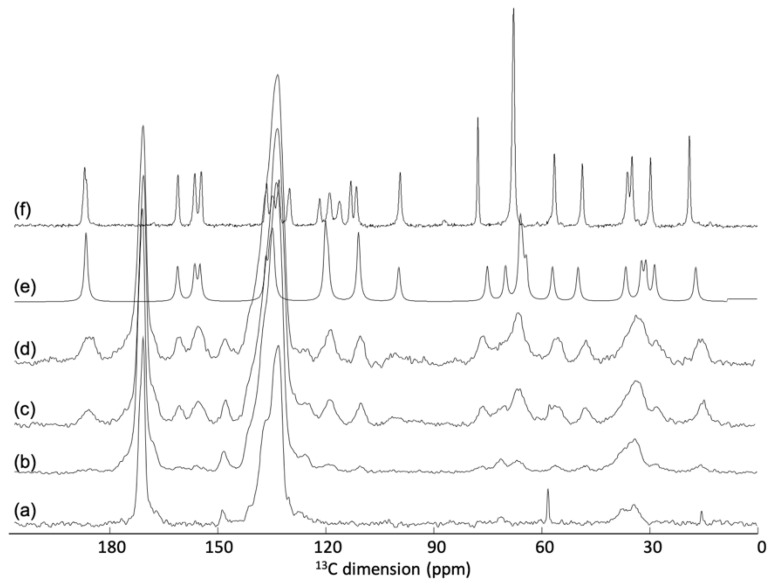
^13^C CPMAS NMR spectra of (**a**) MIL-100(Al) and MIL-100(Al) loaded with (**b**) 8.3 wt.% DOX, (**c**) 14.8 wt.% DOX and (**d**) 27.4 wt.% DOX. In (**f**) is shown the ^13^C NMR spectrum of DOX in the solid-state and in (**e**) a simulation of the liquid-state NMR spectrum.

In an attempt to gain an understanding at the molecular level of the DOX loading processes, a ssNMR investigation on nanoMIL-100(Al) was undertaken. Indeed, ssNMR spectroscopy is well-known to provide atomic-level information about porous MOFs as drug delivery systems [4,5,6,9,10,31]. The ^13^C cross-polarization under magic-angle spinning (CPMAS) NMR spectrum of DOX loaded nanoMIL-100(Al) shows the presence of the DOX molecules (Figure 4). One can notice differences between the position of some carbon resonances of the DOX loaded compared to those of pure crystalline DOX (Figure 4f). It turns out that the position of the loaded DOX is very similar to the ^13^C chemical shift of DOX in solution (Figure 4e), notably in the 110–140 ppm region. The difference between DOX molecules in the crystalline phase and in solution is the presence of the π–π interaction in the crystal that generates particular ^13^C shifts in the aromatic region. The fact that the DOX molecules loaded in the nanoMOFs have chemical shifts similar to that in solution indicates the absence of π–π stacking once incorporated in the MOF suggesting that the drug molecules are incorporated in a molecular state.

MIL-100(Al) particles were loaded with increasing amount of DOX, from 5 to 27 wt.%. While a clear increase of the ^13^C DOX resonance intensity is observed between the 5 and 14.8 wt.% loading (Figure 2b,c), no significant difference is observed between 14.8 and 27.4 wt.% loading (Figure 4c,d). This very likely shows that the amount of DOX that can be loaded inside the pores of the MOF is limited to around 15 wt.%. One possible explanation for the fact that the ^13^C signal of the excess DOX molecules is not detected could be an accumulation of DOX molecules on the surface of the particles. If this external layer of DOX is amorphous, it will generate very broad ^13^C resonances that could be difficult to detect because lying under the peaks of the crystalline phase.

### 3.2. DOX Encapsulation in nanoMIL-100(Al) Coated with CD-CO

#### 3.2.1. Surface Functionalization of nanoMOFs with CD-CO

Given that the citrate moieties could efficiently interact with nanoMOFs [19] and that γ-CD has high affinity with DOX [20], the biodegradable CD-CO was chosen to functionalize nanoMIL-100 (Al). It was successfully synthesized with a yield of around 30% ± 3% using our previous methods [19]. After further characterization using ^1^H NMR, FTIR, and SEC, it was found that the obtained CD-CO possessed an average molecular weight of 22,000 g/mol and a polydispersity (D) of 1.6, in agreement with our previous investigations [19]. Similar results were obtained for CD-^13^CO, which exhibited Mn of 22,800 g/mol (Ð = 1.8) and a γ-CD content of 71 wt.%. This was done employing 1,5-^13^C_2_-citric acid in the polymer synthesis. Preparation of the labeled polymer was successful, as attested by the ^13^C CPMAS NMR spectrum that is similar to the non-labeled polymer (Figure S2). Taking advantage of the affinity between citrate moieties in CD-CO and the Al sites in the nanoMOFs, nanoMOFs were easily functionalized with CD-CO by incubation in water at room temperature. The associated CD-CO amount was quantified by TGA, indicating that the associated CD-CO represented 8 ± 4 wt.% of the initial nanoMOFs amount (Figure S3). The resulting CD-CO@nanoMOFs maintained their facetted structures (Figure 1B), crystallinity (Figure 3A) and BET surface area was only slightly modified (1720 ± 65 m^2^/g and 1500 ± 130 m^2^/g for nanoMOFs before and after surface modification, Figure 3B). This suggests that the coating material does not penetrate inside the MOF porosity. Further evidence of the presence of the coating was given by Zeta potential values, which shifted to negative values in all the studied pH range (1–10). Moreover, the presence of the coating improved the nanoMOF stability as shown in Figure 3C. Without coating nanoMOFs rapidly aggregated whereas with CD-CO shells, their main diameters remained constant over three days’ incubation (less than 5% variations).

#### 3.2.2. Interaction between DOX and CD-CO

**Figure 5:**
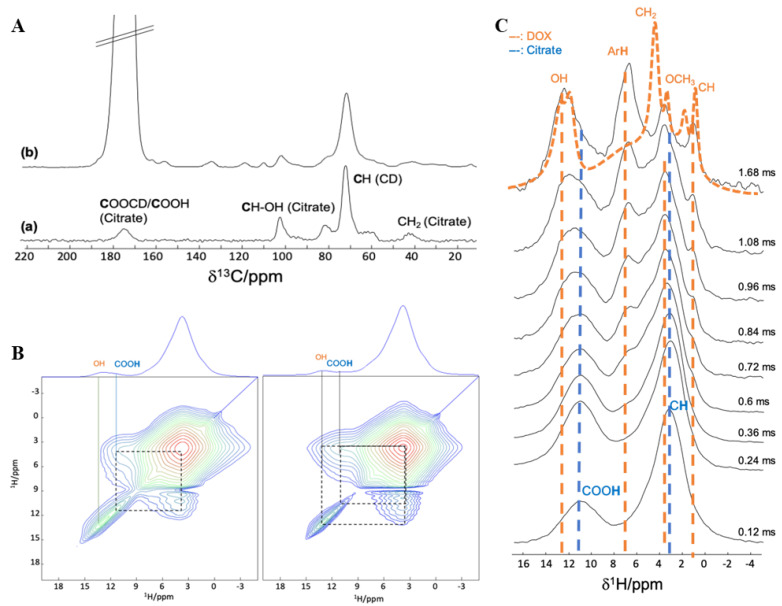
NMR spectra of CD-CO and DOX loaded CD-CO polymer. (**A**). ^13^C CPMAS NMR spectra of CD-CO and DOX loaded CD-^13^CO polymer. Small unlabeled lines correspond to the DOX signals. The strong signal of the labeled ^13^C citrate is truncated for sake of clarity. (**B**). ^1^H-^1^H 2D MAS NMR spectra of DOX loaded CD-CO polymer recorded with mixing time of 5 ms (left) and 15 ms (right) (**C**). ^13^C{^1^H} *D*-HMQC NMR spectrum of DOX loaded CD-CO polymer, recorded for recoupling times ranging from 0.12 to 1.68 ms. For comparison, the ^1^H MAS NMR spectrum of DOX loaded CD-CO is shown in orange dash line. The vertical blue dash lines indicate the position of ^13^C resonances from the CD-CO moieties, while the vertical orange dash lines indicate those of the DOX molecules.

Prior to loading DOX in CD-CO@nanoMOFs, the interaction between CD-CO and DOX was investigated by NMR. ^1^H and ^13^C MAS NMR spectra were recorded on the CD-CO citrate polymer, in order to assess the affinity of DOX with the CD-CO polymer. The ^1^H MAS NMR spectrum (Figure 5B,C) was dominated by the broad peak of the CH_2_ protons of the CD. The ^13^C CPMAS NMR spectrum contains the ^13^C resonances of both the CD (60–80 ppm region) and citrate (carboxyl atoms in the 180 ppm region, CH_2_ around 40 ppm and CH-OH around 100 ppm) moieties (Figure 5A(a)). Note that if the ^13^C resonances of the free COOH and the ester COOCD have similar chemical shifts, a single broad resonance is observed. In order to be able to run 2D ^1^H-^13^C NMR experiments and extract more information about CD-CO and DOX interactions, we chose to enrich the citrate moieties with ^13^C isotope (since ^13^C natural abundance is too low at ca 1%). This choice of isotopic enrichment of the coating only (and not of the MOF linker) provides higher selectivity on the coating–MOF surface interactions of prime interest in this study.

Incorporation of the DOX molecules in the ^13^C-labeled polymer was also achieved and at DOX loading of 14.8 wt.%, all ^13^C resonances of the DOX are observed (Figure 5A(b)). Although the resonances were still very broad and partially overlapping, some resolution was obtained in the ^1^H MAS NMR spectrum of CD-CO-DOX, notably in the aromatic and OH region of the DOX (above 7 ppm). ^1^H-^1^H 2D MAS NMR experiments were carried out (Figure 5B) to probe spatial proximities between the protons of the CD-CO and those of the DOX. At shorter recoupling time (left spectrum), the ^1^H-^1^H NMR spectra shows as expected cross-correlation peaks between the unconnected COOH of the citric moieties (11 ppm) and the CH/CH_2_ protons of the CO and CD (broad peak around 4 ppm). For longer recoupling time (right spectrum), one can also observe a cross-correlation peak between this CH/CH_2_ peak and the peak corresponding to the OH of the DOX molecules, indicating close contact between the DOX and the CD-CO inside the particles.

Taking advantage of the ^13^C-tag of the citrate moieties (that significantly increases the sensitivity of ^13^C experiment), we also probed ^13^C–^1^H proximity. In this experiment, the magnetization of the ^13^C-citrate was selectively chosen and transferred to its surrounding protons. The intensity of the resulting proton resonances as a function of transfer time is shown in Figure 5C. Short ^13^C-carbon-proton spatial proximities are dominant at a short recoupling time, while longer C–H distances appear at longer transfer time. In this figure, at a short recoupling time, mostly the protons of the CD and citrate moieties are seen, as expected since the ^13^COO belong to the citrate polymer. For longer magnetization transfer time, the protons of the DOX start also to be observed. This confirms the close mixing of the DOX molecules within the CD-CO polymer. Interestingly, the aromatic protons of the DOX have much higher intensity on the ^1^H NMR spectrum after transfer from the ^13^C-citrate than in the normal ^1^H MAS NMR spectrum (orange spectrum on Figure 5C). This indicates that the aromatic parts of the DOX molecules have a preferential interaction with the ^13^CD-CO polymer.

#### 3.2.3. Characterization of DOX Loaded MIL-100(Al) nanoMOFs Coated with CD-CO

DOX payload: Due to this strong binding of DOX to CD-CO [20] a direct quantification of DOX in the supernatant was not possible by fluorescence measurements. Thus, a method was set up to quantify DOX, based on disruption of the inclusion complexes by adding DMSO (see the Materials and Methods). In these conditions, DOX fluorescence intensity was not influenced by the presence of CD-CO.

Interestingly, as shown in Figure 2, the presence of the CD-CO coatings accelerated the loading process. For example, at TDL of 20%, DOX payloads reached 11.5 ± 0.8 wt.% and 12.8 ± 0.7 wt.% for bare nanoMOFs and CD-CO@nanoMOFs, respectively, after 2 days’ incubation (Figure 1A). This could be explained by the fact that DOX has a high affinity for both CD-CO^20^ and MIL-100 (Al) nanoMOFs [13]. After 6 days’ impregnation, maximal drug loading reached 14.8 ± 1.0 wt.% and 15.3 ± 0.8 wt.% for bare nanoMOFs and CD-CO@nanoMOFs, respectively (Figure 2A). Noticeably, EE reached more than 73.9% after 6 days for all the cases. When excess DOX was added in the system (TDL of 50%), DL reached 27.4 ± 2.0 wt.% and 27.8 ± 0.8 wt.% for bare nanoMOFs and CD-CO@nanoMOFs, respectively. Therefore, the CD-CO coating did not significantly change the DOX payload, but it could accelerate the loading process. For example, in the case of TDL of 50%, DOX payload of 16 ± 2.4 wt.% and 19 ± 1.9 wt.% for nanoMOFs and CD-CO@nanoMOFs was observed after 1 day of incubation.

After both the DOX loading and CD-CO coating, the crystallinity of the particles was still preserved with slight long-range changes (Figure 3A). Zeta potential was shifted to negative values compared to the empty nanoMOFs, indicating that the CD-CO coating played a major role on the surface charge, which masked the effect of DOX molecules loaded within the pores close to the external surface.

**Figure 6:**
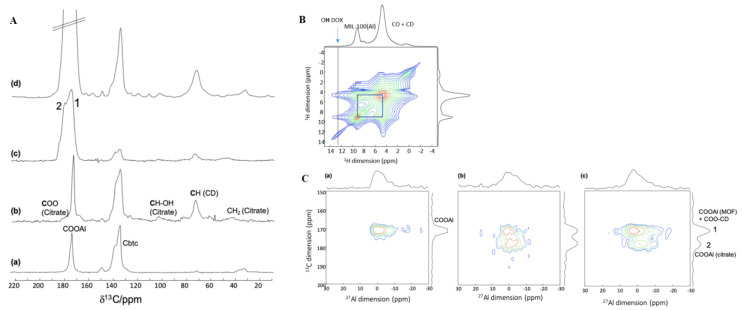
NMR spectra of MIL-100(Al), CD-CO@nanoMIL-100(Al), CD-^13^CO@nanoMIL-100(Al), and DOX loaded CD-^13^CO@nanoMIL-100(Al). (**A**). ^13^C CPMAS NMR spectra of (a) MIL-100(Al), (b) CD-CO@nanoMIL-100(Al), (c) CD-^13^CO@nanoMIL-100(Al), and (d) DOX loaded CD-^13^CO@nanoMIL-100(Al). In (d), the small unlabeled peaks correspond to the DOX molecules. The intense ^13^C resonances is truncated for sake of clarity. In (c), 1 and 2 show the two labeled carboxylic resonances. Line 2 corresponds to the ^13^C carboxylic atoms of the CO linked to the surface aluminum sites of the MOF. Line 1 is a superimposition of COOAl carboxylic of the trimesate BTC linker and the ^13^COO-CD of the polymer coating. (**B**). ^1^H-^1^H 2D MAS NMR spectrum of DOX loaded CD-^13^CO@nanoMIL-100(Al) dried. (**C**). 2D ^13^C-^27^Al *D*-HMQC 2D of (a) nanoMIL-100(Al), (b) CD-^13^CO@nanoMIL-100(Al) and (c) DOX loaded CD-^13^CO@nanoMIL-100(Al).

To understand the affinity between the covering CD-CO oligomers and the MOF surface, ^1^H, ^27^Al, and ^13^C MAS NMR were recorded on MIL-100(Al) nanoMOFs and CD-CO@nanoMIL-100(Al). The ^27^Al MAS NMR of both samples is similar (Figure S4), with all Al atoms in a six-fold coordination. In the ^13^C CPMAS NMR spectra (Figure 6A), the ^13^C resonances corresponding to the CD-CO could be identified. Note an overlap of the carboxylic ^13^C resonances with those of the carboxylic carbon atoms from the MOF linker in the 170–180 ppm region. Due to the small proportion of coating with respect to the nanoMOF size, the signals of the CD-CO had low intensity and it was therefore difficult to distinguish between the ester (CO-CD) and the free COOH of the citric acid moieties.

^1^H MAS NMR spectra were also recorded (Figure 6B). However, as was already observed for MIL-100(Al) nanoMOFs coated with CD-P [17], due to the presence of numerous protons both in the MOF and in the coating, no useful information could be extracted. In CD-P coated MIL-100(Al) NPs, this difficulty could be circumvented by using the heteroatoms present either in the MOF only (i.e., ^27^Al) or in the coating only (^31^P). This allowed clear probing of the coating-MOF interactions. In the system under study here, while the MOF still contains a heteroatom (^27^Al), the coating only contains ^1^H and non-abundant ^13^C (ca. 1% natural abundance). Therefore, we chose to take advantage of the ^13^C-labeled CD-CO polymer presented in the previous section. This way, ^27^Al was selectively present in the MOF, while ^13^C nucleus was present in significant quantity both in the coating (labeled ^13^C) and in the nanoMOFs (natural abundance ^13^C of the linker).

^13^C CPMAS NMR spectra of the nanoMOFs coated with non-labeled or labeled CD-CO polymers are similar (Figure S2). No significant difference in the ^1^H and ^27^Al ssNMR spectra is noticed with the unlabeled product (Figure S5). With the higher intensity provided by the ^13^C labeling, the carboxylic groups of the citric acid are now very visible. In particular, contrary to the CD-CO polymer shown in the previous section, two resonances, labeled 1 and 2 in Figure 6A(c), for the ^13^C-labeled carboxylic carbon atoms of the citric moieties are observed. Since both the ^13^COO-CD ester and the free ^13^COOH had similar chemical shift in the pure polymer, one can infer that the new peak observed in the CD-CO@nanoMIL-100(Al) arises from a strong interaction (maybe a chemical bond) between the citrate and the Al sites located at the surface of the nanoMOF. Such a strong interaction was already observed in the case of CD-P coating [17]. Note the absence of the COOH proton signal in the ^1^H MAS NMR spectrum, which further support the formation of a bond between the free COOH of the CD-CO and the Al atoms located at the surface of the nanoMOF.

To understand better the interaction between the CD-CO coating and the nanoMOFs, 2D ^13^C-^27^Al MAS correlation NMR experiments were performed (Figure 6C). These experiments show the spatial proximity between carbon and aluminum atoms. Correlation peaks of strong intensity are observed between the CD-CO and the surface Al sites, confirming that the CD-CO polymer had high affinity with the NP surface. Note that the ^13^C resonance at 175 ppm contains both the carboxylic group of the trimesate linker of the MOF (not labeled but present in large quantity) and the COO-CD of the CD-^13^CO coating. ^13^C-^27^Al double-resonance curves were recorded (Figure S6), which give indication on relative C-Al distances. These curves show that the peak at 180 ppm (line 2) has shorter distance to the Al than the peak at 175 ppm (line 1). This supports further the hypothesis that this additional peak results from a chemical bond formed between some of the free COOH groups of the CD-CO polymers and the Al atoms located at the surface of the nanoMOFs.

In the DOX loaded CD-^13^CO@nanoMIL-100(Al), the ^13^C signals of the DOX molecules are seen and are similar to the solution state ^13^C NMR spectrum of DOX, indicating the absence of π–π interactions (Figure 6A(d)). The ^13^C-^27^Al 2D MAS NMR spectrum (Figure 6C(c)) shows that the coating and the NPs are still in a strong interaction. However, the relative intensity between the ^13^CO peaks 1 and 2 is different. This indicates that, in addition to going in the pores of the MOF as shown earlier, the DOX molecules also interact significantly with the CD-CO coating. Notably, since the intensity of ^13^C line labeled 2 has decreased, it very likely indicates that part of the ^13^COO-Al bonds formed between the citric acid moieties and the surface Al sites have been broken after DOX incorporation.

### 3.3. DOX Release Studies

**Figure 7:**
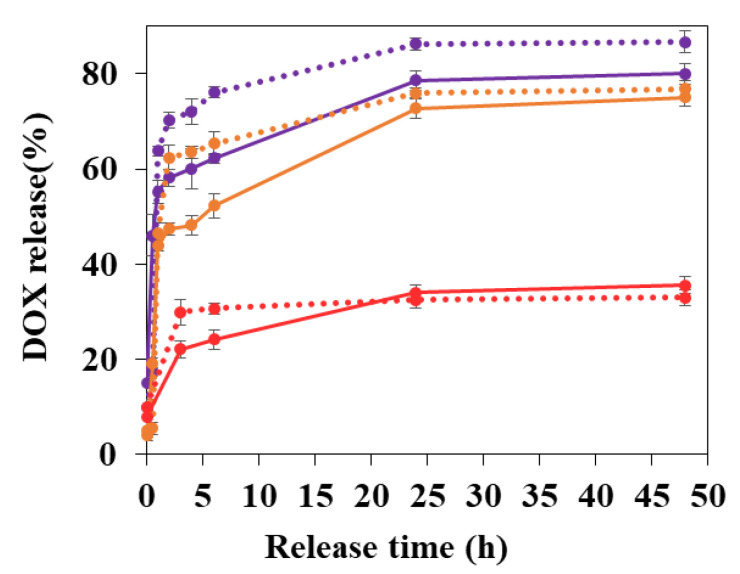
DOX release in PBS (pH 7.4, 9.5 mM, 37 °C). DOX loaded nanoMOFs (continuous line) and DOX loaded CD-CO@nanoMOFs (dashed line) at different TDLs (purple: 10%; orange: 20%; red: 50%).

DOX release profiles in PBS at 37 °C are shown in Figure 7. At each time point, DOX released in the supernatant was recovered after centrifugation and quantified by fluorescence spectroscopy. The method was validated by DOX quantification in the pellets. To do so, several solvents and degrading agents were used. PBS was used previously in our teams [38] to extract drugs from nanoMOFs by degrading them completely, which released both the timesate linkers and the drugs. However, this method was unsuccessful in the case of DOX-loaded nanoMOFs, most probably because of the strong affinity of DOX for these matrices. Eventually, deferoxamine mesylate salt was used to erode the MOFs and extract all their drug content. Mass balance studies showed recoveries of 95% ± 5.5%.

When DOX was loaded at TDL of 20%, DOX release from bare nanoMOFs reached around 43.8% ± 1.2% within 1 h and 75.1% ± 2.0% in 2 days (Figure 7).

Interestingly, the DOX release behavior was found to be dependent on the DOX payloads. When less DOX was loaded, in the case of DOX payload of 8.3 ± 0.7 wt.%, more DOX was released in the same condition (60.1% ± 4.1% and 48.2% ± 2.8% in 4 h for TDL of 10% and 20%, respectively). At high DOX loading (TDL of 50%), there was only 35.6% ± 1.9% released out in 2 days. In other words, at high DOX contents in the nanoMOFs, not all the drug is released out. Possibly, DOX self-associates inside the cages leading to incomplete release. This phenomenon has been already shown with another drug, which tends to self-associate, topotecan, which was incompletely released from MIL-100 (Fe) nanoMOFs [39].

In a nutshell, it appears that the advantage of CD coatings is to better extract DOX from the cores, especially at early times (<6 h) and for low DOX payloads (especially TDL of 10%). CD coating accelerates the loading process and facilitates DOX release.

## 4. Conclusions

In this study, we reported the synthesis and characterization of CD-CO coated nanoMIL-100(Al) particles loaded with DOX molecules. Due to the high affinity of DOX with both the CD-CO coating and the MOF, a high amount of drug could be loaded in the nanoparticles. High-resolution ssNMR spectroscopy was employed to characterize the core–shell nanoMOFs. In order to perform informative ^1^H-^13^C and ^27^Al-^13^C NMR experiments, the key element was to synthesize ^13^C-labeled CD-CO oligomers, which were coated on the nanoMOF external surface. The ensemble of NMR data unambiguously confirms the high affinity of DOX with both the CD-CO moieties and the nanoMOF. Interestingly, it was found that the DOX release was dependent on the initial DOX loading rate. This finding indicates that CD coatings were promising to accelerate the loading process and facilitates DOX release.

The ssNMR methodology associated to the selective isotope labeling strategy proved efficient for the characterization of this system based on diamagnetic nanoMOF. With further technical developments and adaptation to paramagnetic system, this approach could be useful to characterize other drug delivery systems such as MIL-100(Fe) nanoMOFs.

## Data Availability

Data is contained within the article or Supplementary Material.

## Supplementary Materials

The following are available online at https://www.mdpi.com/article/10.3390/nano11040945/s1, Figure S1: FTIR Characterization of the particles before and after drug loading and/or surface functionalization (blue: empty nanoMOFs; dark green: CD-CO; green: nanoMOFs coated with CD-CO; red: nanoMOFs loaded with DOX at TDL of 50%; red dashed line: nanoMOFs loaded with DOX at TDL of 50% and coated with CD-CO). Figure S2: ^13^C CPMAS NMR spectra of (a) CD-CO coated nanoMIL-100(Al) and (b) 13C-CD-CO coated nanoMIL-100(Al), Figure S3: TGA curve of nanoMOFs (blue) and CD-CO coated nanoMOFs (green), Figure S4: ^27^Al MAS NRM spectrum of nanoMIL-100 (bottom) and CD-CO coated nanoMIL-100 (top), Figure S5: ^1^H (left) and ^27^Al (right) MAS NMR spectra of (a) CD-CO coated nanoMIL-100(Al) and (b) ^13^C- CD-CO coated nanoMIL-100(Al), Figure S6: Left: ^13^C-^27^Al CP-RESPDOR NMR spectra (left) with non (green) and saturation (blue) of Al nuclei, recorded at 12.5 kHz (9.4 T) at different recoupling time. Right: RESPDOR curves of Line 2 (corresponding to COOAl (CO) and shown in orange) and Line 1 (corresponding to COOAl (MIL-100(Al)) + COOCD (CO) and shown blue).
