# Supplementary material for: Doxorubicin-Loaded Metal-Organic Frameworks Nanoparticles with Engineered Cyclodextrin Coatings: Insights on Drug Location by Solid State NMR Spectroscopy
Source: Nanomaterials (Basel). 2021 Apr 8;11(4):945. doi: 10.3390/nano11040945 (PMC8068177; doi:10.3390/nano11040945)
Supplement: Supplementary file 1 [file nanomaterials-11-00945-s001.pdf]

Supporting information

Doxorubicin-Loaded Metal-Organic Frameworks Nanoparticles with Engineered Cyclodextrin Coatings: Insights on Drug Location by Solid State NMR Spectroscopy

Xue Li [1,†], Marianna Porcino [2,†], Jingwen Qiu [1], Doru Constantin [3], Charlotte Martineau-Corcos [2,4,*,‡] and Ruxandra Gref [1,*]

[1] Institut des Sciences Moléculaires d'Orsay, UMR CNRS 8214, Paris-Sud University, Université Paris Saclay, 91400 Orsay, France; xue.li@universite-paris-saclay.fr (X.L.); jingwen.qiu@universite-paris-saclay.fr (J.Q.)

[2] CEMHTI UPR CNRS 3079, Université d'Orléans, F-45071 Orléans, France; marianna.porcino@cnrs-orleans.fr

[3] Laboratoire de Physique des Solides, UMR 8502, Université Paris-Sud, 91405 Orsay, France; doru.constantin@universite-paris-saclay.fr

[4] Université Paris Saclay, ILV UMR CNRS 8180, Université de Versailles St-Quentin en Yvelines, 78035 Versailles, France

* Correspondence: ccorcos@cortecnet.com (C.M.-C.); ruxandra.gref@universite-paris-saclay.fr (R.G.); Tel.: +33-(0)-1-30-12-11-31 (C.M.-C.); +33-(0)-1-69-15-82-34 (R.G.)

† These authors contributed equally.

‡ Current Address: CortecNet, 7 avenue du Hoggar, 91940 Les Ulis, France.

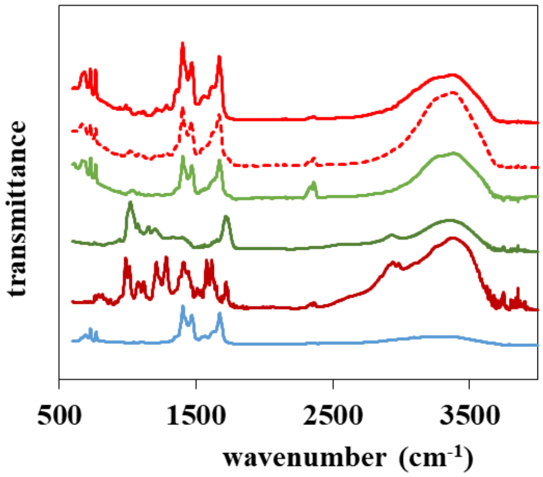

Figure S1. FTIR Characterization of the particles before and after drug loading and/or surface functionalization (blue: empty nanoMOFs; dark green: CD-CO; green: nanoMOFs coated with CD-CO; red: nanoMOFs loaded with DOX at TDL of 50 %; red dashed line: nanoMOFs loaded with DOX at TDL of 50% and coated with CD-CO).

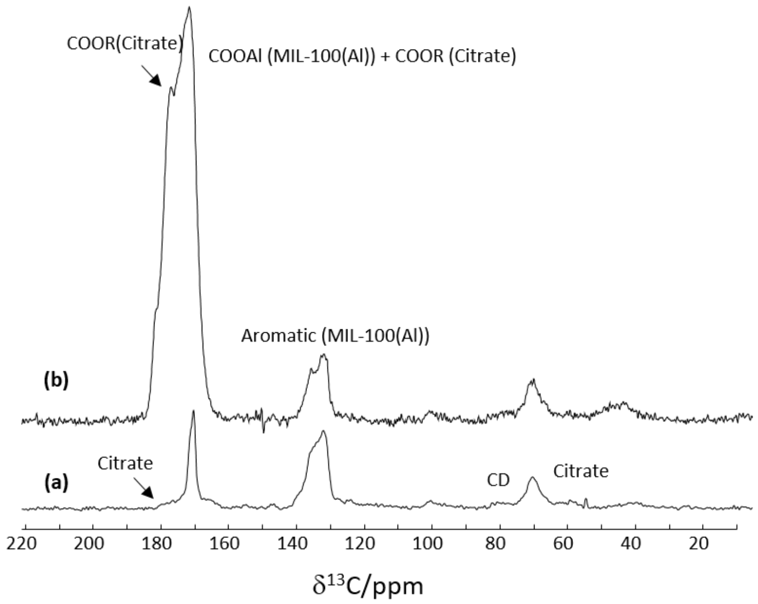

Figure S2. ^{13}C CPMAS NMR spectra of (**a**) CD-CO coated nanoMIL-100(Al) and (**b**) ^{13}C-CD-CO coated nanoMIL-100(Al)

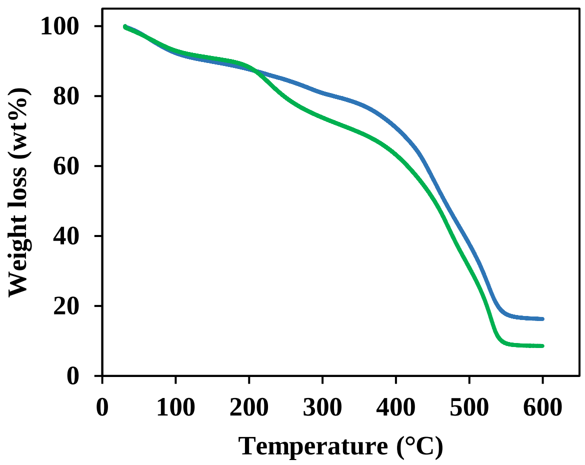

Figure S3. TGA curve of nanoMOFs (blue) and CD-CO coated nanoMOFs (green)

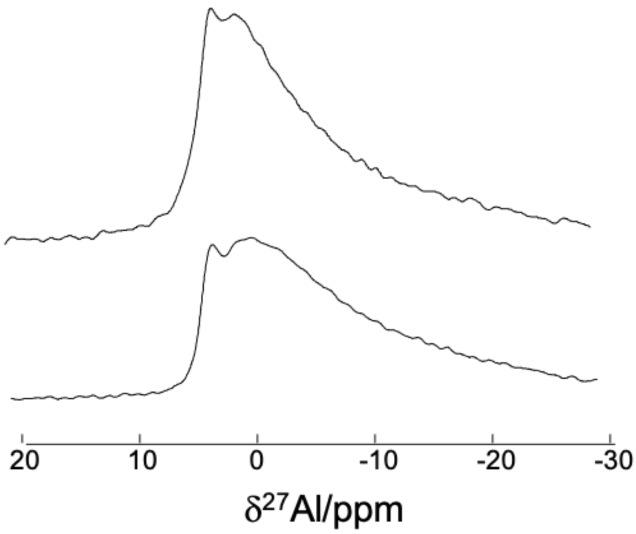

Figure S4. ^{27}Al MAS NMR spectrum of nanoMIL-100 (bottom) and CD-COcoated nanoMIL-100 (top)

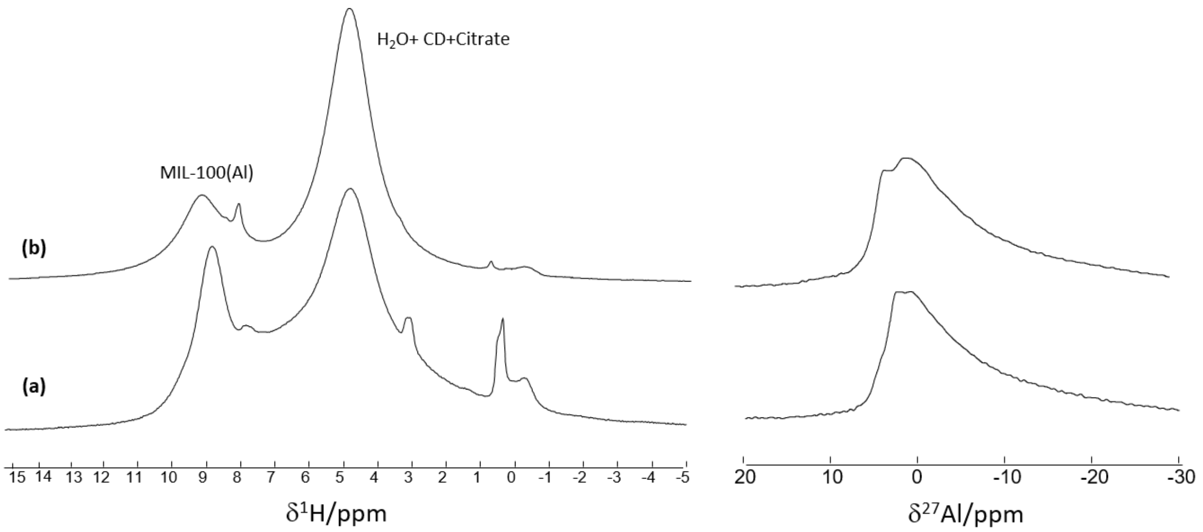

Figure S5. ^{1}H (left) and ^{27}Al (right) MAS NMR spectra of (a) CD-CO coated nanoMIL-100(Al) and (b) ^{13}C- CD-CO coated nanoMIL-100(Al)

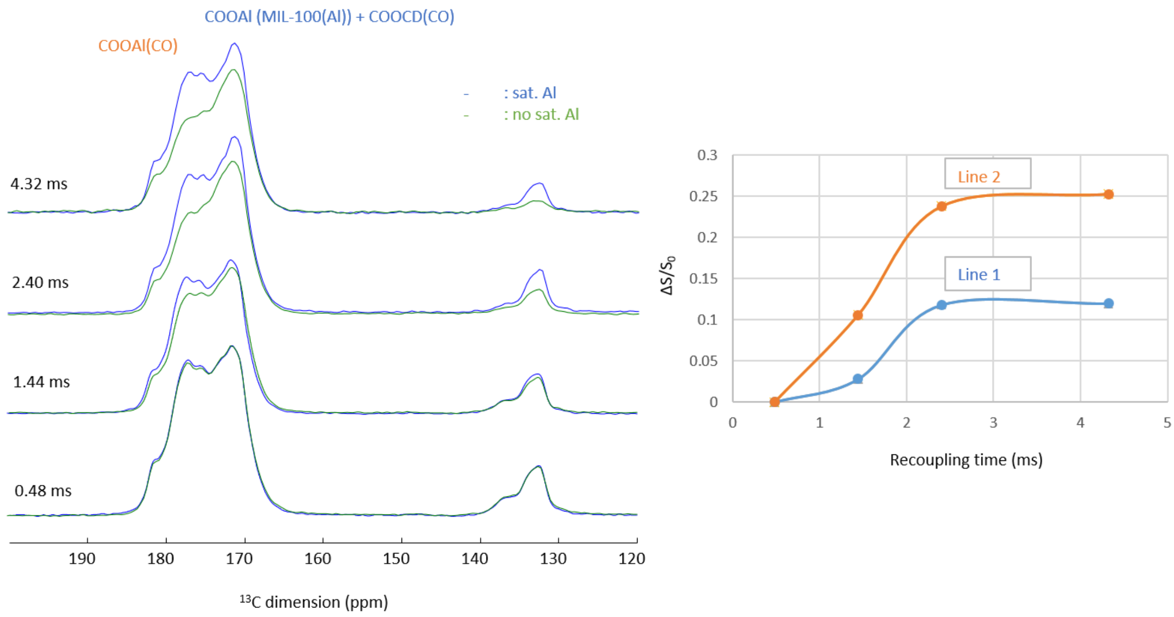

Figure S6. Left: ^{13}C-^{27}Al CP-RESPDOR NMR spectra (left) with non (green) and saturation (blue) of Al nuclei, recorded at 12.5 kHz (9.4 T) at different recoupling time. Right: RESPDOR curves of Line 2 (corresponding to COOAl (CO) and shown in orange) and Line 1 (corresponding to COOAl (MIL-100(Al)) + COOCD (CO) and shown blue)
